# Transversus abdominis plane block following abdominally based breast reconstruction: study protocol for a randomized controlled trial

**DOI:** 10.1186/1745-6215-14-424

**Published:** 2013-12-10

**Authors:** Toni Zhong, Marie Ojha, Shaghayegh Bagher, Kate Butler, Anne C O’Neill, Stuart A McCluskey, Hance Clarke, Stefan OP Hofer, Coimbatore Srinivas

**Affiliations:** 1 Division of Plastic & Reconstructive Surgery, University Health Network, 200 Elizabeth St, Toronto, ON M5G 2C4, Canada; 2 Division of Plastic and Reconstructive Surgery, University of Toronto, 149 College Street, Toronto, ON M5T 1P5, Canada; 3 Adult Acute Pain Service, University Health Network, 200 Elizabeth St., Toronto, ON M5G 2C4, Canada; 4 Division of Anesthesia and Pain Management, University Health Network, 200 Elizabeth St., Toronto, ON M5G 2C4, Canada

**Keywords:** Autologous abdominal tissue, Breast reconstruction, Donor site, Local pain block, Transversus abdominis plane catheter

## Abstract

**Background:**

Breast reconstruction using the free muscle-sparing transversus abdominus myocutaneous or deep inferior epigastric perforator flaps are common methods for restoring mastectomy defects for breast cancer patients. Despite its increasing popularity and safety, the abdominal donor site remains a major source of postoperative pain. Conventional postoperative pain relief protocol consists primarily of a patient-controlled anesthesia device delivering intravenous opioids. Opioids can cause numerous side effects such as sedation, headache, nausea, vomiting, breathing difficulties and bladder and bowel dysfunction. A promising approach to provide postoperative pain control of the abdominal incision is the newly developed transversus abdominis plane peripheral nerve block.

**Methods/Design:**

This study is a double-blind, placebo-controlled, randomized controlled trial designed to rigorously test the effectiveness of a transversus abdominis plane catheter delivering intermittent local anesthetic in reducing postoperative abdominal pain following abdominal tissue breast reconstruction. The primary objective of this study is compare the mean total opioid consumption in the first postoperative 48 hours between the control and study groups including the patient-controlled anesthesia amounts and oral narcotic doses converted to intravenous morphine equivalent units. The secondary outcome measures include the following parameters: total in-hospital cumulative opioid consumption; daily patient-reported pain scores; total in-hospital cumulative anti-nausea consumption; nausea and sedation scores; and Quality of Recovery score; time to first bowel movement, ambulation, and duration of hospital stay.

**Discussion:**

Autologous breast reconstruction using abdominal tissue is rapidly becoming the reconstructive option of choice for postmastectomy patients across North America. A substantial component of the pain experienced by patients after this abdominally based procedure is derived from the abdominal wall incision. By potentially decreasing the need for systemic opioids and their associated side effects, this transversus abdominis plane block study will utilize the most scientifically rigorous double-blind, placebo-controlled, randomized controlled trial methodology to potentially improve both clinical care and health outcomes in breast cancer surgery patients.

**Trial registration:**

Clinicaltrials.gov NCT01398982

## Background

Autologous breast reconstruction using abdominal tissue is rapidly becoming the reconstructive option of choice for postmastectomy patients across North America. A substantial component of the pain experienced by patients after abdominally-based autologous tissue breast reconstruction is derived from the abdominal wall incision [1-3]. The conventional postoperative analgesic regimen following abdominally based autologous tissue breast reconstruction still primarily utilizes systemic opioids, which can cause sedation, headache, nausea, vomiting, respiratory compromise, pruritus and bladder and bowel dysfunction [4,5].

It is well-known that adequate analgesia decreases the incidence of cardiopulmonary complications and in-hospital deaths [6]. Several authors have investigated methods of improving postoperative analgesia with the use of a local anaesthetic (LA) infusion catheter in the donor site following abdominal flap reconstruction of the breast [7-11]. To date, the results have been mixed in these studies aimed at decreasing pain in the donor site following free muscle-sparing transversus abdominus myocutaneous (MS-TRAM) flaps or deep inferior epigastric perforator (DIEP) flaps [12-16]. The use of peripheral nerve blocks has become increasingly popular over the past two decades. With the use of anatomical landmark-based techniques, novel types of nerve blocks have been designed to deliver effective and directed analgesia. The transversus abdominis plane (TAP) block is a newly developed block involving the T6-L1 intercostal nerves, which supply the anterior abdominal wall [17-20]. In the TAP block, the lumbar triangle of Petit is used as a landmark for injecting LA into the neurovascular plane of the abdominal wall (the TAP plane) between the internal oblique and transversus abdominis muscle layers [17]. The triangle of Petit is configured with the iliac crest forming the base, the external oblique muscle being the anterior border and the latissimus dorsi muscle being the posterior border of the triangle. The floor of the triangle is made up of superficial fascia covering the transversus abdominis muscle. Both the blind TAP block and ultrasound-guided TAP block have been associated with minimal complications [7,8,11-13,15]. Recent published clinical trials involving patients who have undergone major abdominal and gynaecological surgery have demonstrated promising results with the use of this technique as part of multimodal postoperative pain treatment [7,8,10,21-24].

Our group recently published the results of a prospective cohort study that compared the total intravenous *patient-controlled analgesia* (PCA) opioid consumption between a group of 45 consecutive patients who received intermittent postoperative bolus injections of 0.25% bupivacaine through the TAP catheter and a group of 80 historic control patients who underwent the same abdominally based microsurgical breast reconstruction without TAP blocks [25]. We found that the 48-hour PCA-delivered opioid requirement was significantly less (*P* < 0.001) in the TAP block group (17.10 ± 17.23 mg intravenous morphine equivalent) compared to the control group (48.44 ± 39.53 mg). Although we concluded on the basis of data derived from our study that intermittent delivery of bupivacaine through the TAP block significantly reduced postoperative parenteral opioid requirements following abdominally based breast reconstruction, there are inherent biases associated with using historic controls. Therefore, we designed our present double-blind, placebo-controlled, randomized controlled trial (RCT) to rigorously assess the efficacy of reducing postoperative donor site pain by intermittently delivering a LA agent through the TAP catheter in patients undergoing breast reconstruction using abdominal tissue microsurgical reconstruction.

## Methods

### Study design

**Figure 1:**
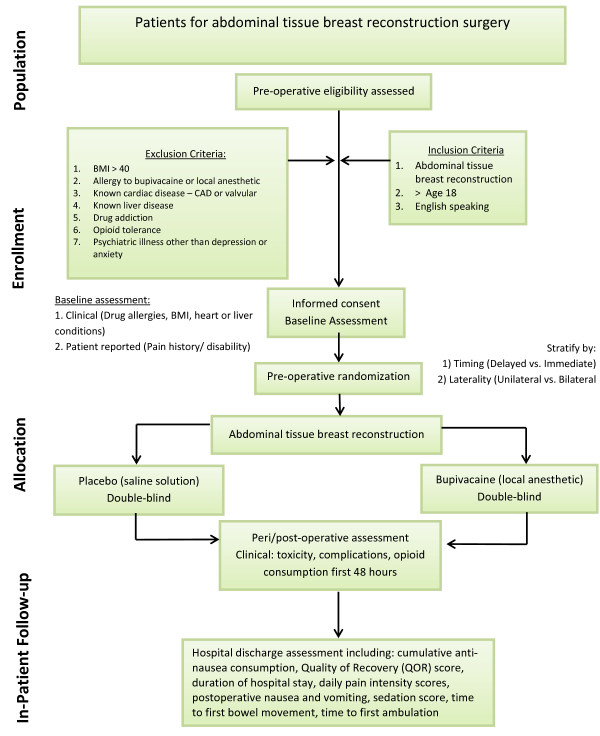
**Transversus abdominis plane trial design chart.** BMI, body mass index.

This double-blind, placebo-controlled RCT is a two-group parallel superiority trial. Figure 1 provides an overview of the trial design. Institutional research ethics board approval was obtained from the University Health Network (REB 10-0969-A). The source population will be members of the mastectomy cohort identified from the Breast Reconstruction Clinic at Toronto General Hospital by the two participating plastic surgeons (TZ and SOPH). Potential research participants will be reviewed by a research coordinator, who will determine the suitability of each patient by reviewing her medical records (see Eligibility). Full written informed consent will be obtained from each participant by the research coordinator.

### Randomization

Once informed consent is obtained from the patient to participate in this trial, the research assistant will contact the study pharmacist to obtain the patient’s randomization arm. Patients will be randomized to receive either bupivacaine (study group) or isotonic saline (control group) through the TAP catheter by intermittent injection. The randomization allocation list will be developed by the Department of Epidemiology and Biostatistics at the University Health Network Research Institute. Randomization allocation will be equal between the study groups and stratified by laterality of abdominal flap harvest (unilateral vs. bilateral) and the timing of breast reconstruction (at the same time as mastectomy (immediate) vs. at a later time, after the mastectomy (delayed)). Sequentially numbered and sealed envelopes containing the treatment assignments will be prepared and given to the study pharmacist. The patient, surgeon, research assistant and all other study personnel will be blinded to randomization. The randomization code will be revealed only after the completion of the trial and all the patient data have been collected.

### Eligibility

The following are the inclusion criteria. (1) Patients must be older than 18 years of age, with no upper age limit. (2) Participants must be able to speak English. (3) Treatment must be immediate or delayed microsurgical breast reconstruction using abdominal tissues (free MS-TRAM or DIEP flap).

The following are the exclusion criteria: (1) patient refusal; (2) inability to give informed consent; (3) body mass index (BMI) greater than 40; (4) allergy to bupivacaine; (5) patients who will undergo implant breast reconstruction or non–abdominally based autologous tissue reconstruction; (6) any drug addiction; (7) opioid tolerance, defined as preoperative daily opioid use higher than 50 mg of a morphine equivalent by mouth (in the previous 2 months); and (8) any psychiatric illness, excluding depression and anxiety.

### Intervention plan

#### Surgical technique: insertion of the transversus abdominus plane catheter

**Figure 2:**
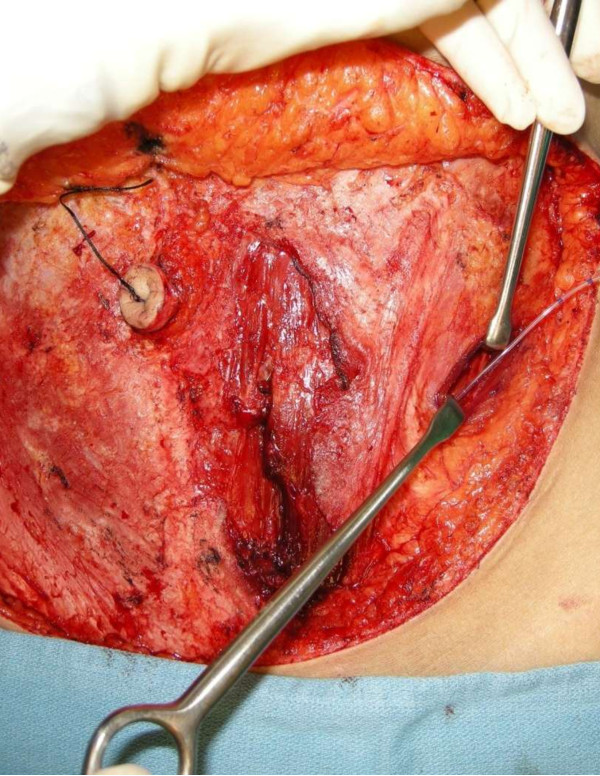
Triangle of Petit landmarks are identified through the same abdominal incision used to harvest the abdominal flap.

The abdominal flaps will be harvested in a standardised manner with preservation of nerves in all cases. In both groups, the plastic surgeon will close the rectus fascia following free DIEP or MS-TRAM harvest in the routine fashion. If a synthetic mesh is required for abdominal closure, the patient will be excluded from the study. The lumbar triangle of Petit will be located by identifying its borders between the latissimus dorsi muscle posteriorly, the external oblique muscle anteriorly and the iliac crest inferiorly. (Figure 2) A 2-cm incision will be made through the abdominal fascia in the triangle of Petit. The catheter will be inserted through a stab incision 5 cm above the lateral edge of the abdominal incision. This catheter will be introduced into the TAP incision and advanced into the transversus abdominis fascial plane. The fascial defect in the triangle of Petit will be repaired using a Polysorb 0 suture (Covidien, Mansfield, MA, USA). The catheter is secured to the skin using OpSite Flexigrid tape (Smith & Nephew, St Petersburg, FL, USA) to prevent accidental dislodgement. In both unilateral and bilateral cases, two catheters will be inserted, one on each side.

#### Intraoperative analgesic protocol

Intraoperatively, fentanyl in aliquots of 1 μg/kg will be administered for pain control, in addition to an induction dose of 3 μg/kg. Upon completion of the surgery, each patient will receive a dose of 50 to 100 mg of indomethacin and 1,300 mg of acetaminophen per rectum. In addition, ketorolac will not be given intraoperatively. Dexamethasone (4 mg) will be administered in the operating room (OR) for all patients to prevent postoperative nausea and vomiting.

#### Intermittent transversus abdominis plane block protocol

**Figure 3:**
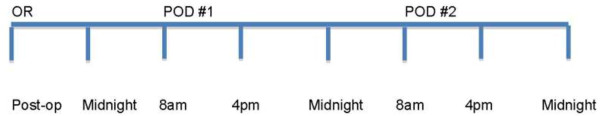
**Intermittent bolus injection diagram.** OR, operating room; POD, postoperative day.

At the conclusion of surgery, a 0.2 ml/kg bolus of 0.25% bupivacaine or saline solution will be injected through each catheter in the OR. At midnight following the operation, 0.2 ml/kg of 0.25% bupivacaine or saline will be injected through each catheter every 8 hours for the next 2 postoperative days by a member of the pain service. At 8 AM on postoperative day 3, the TAP catheters will be removed by the pain team (Figure 3).

#### Local anesthetic toxicity

For a 70-kg female undergoing bilateral breast reconstruction, the use of 0.2 ml/kg of 0.25% bupivacaine in each TAP catheter every 8 hours in the study group will result in a 1 mg/kg dosage bupivacaine ((0.2 ml/kg × 70 kg × 2 sides = 28 ml) (70 mg × 0.25% concentration = 70 mg)). Because the maximum recommended dosage of bupivacaine over 6 hours is 2.5 to 3.0 mg/kg, our prescribed dosing protocol every 8 hours is well below the toxic range.

#### Postoperative treatment

Each patient will receive intravenous hydromorphone through a PCA pump programmed for demand-only mode with no basal rate. PCA will be set at 0.1 mg per bolus every 5 minutes as needed (to a maximum of 10 mg per 4 hours), which will be initiated in the recovery room. The PCA will be filled with hydromorphone as per standard care at our institution. When patients can tolerate liquids, they will be given 1 g of plain acetaminophen every 6 hours around the clock. Once the PCA is discontinued, patients will be offered 5 to 10 mg of oxycodone every 2 to 4 hours as required, in addition to 1 g of acetaminophen every 6 hours around the clock. Granisetron (1 mg every 12 hours will be the first-line therapy for postoperative nausea and vomiting. Patients will be encouraged to ambulate on postoperative day 1. A liquid diet, then a solid diet, will be offered on postoperative day 1 and advanced as tolerated. The PCA system will be discontinued at 8 AM on postoperative day 3.

### Primary end point and secondary end points

The primary end point evaluated will be the mean postoperative opioid consumption in the first 2 postoperative days. Both intravenous PCA opioids and oral opioids will be recorded from the patients’ medical records daily. All opioid doses will be converted to intravenous morphine equivalent units for comparison.

The secondary outcomes of interest will include the following. The continuous outcomes will be total in-hospital cumulative opioid consumption, total in-hospital cumulative antinausea medication consumption, score on the Quality of Recovery (QoR) questionnaire administered once on postoperative day 4 (scored from 0 to 18, with a greater score indicating better QoR) [9] and duration of hospital stay. Therepeated-measures outcomes will be assessed by recording daily pain intensity scores at rest and with movement measured using a Visual Analogue Scale for pain (scored from 0 to 10, with 0 indicating no pain and 10 indicating worst pain ever), postoperative nausea and vomiting (scored from 0 to 3) and sedation score. The time to event outcomes will be recorded for time to first postoperative bowel movement and time to ambulation.

The data extraction forms will be completed prospectively by a member of the pain team.

### Data analysis

Patients who have major postoperative complications that require a second surgery, such as evacuation of a hematoma or microsurgical revision, will not be removed from the study and will be evaluated using the intention-to-treat analysis. An initial examination will be performed to detect any differences between the two treatment groups despite the randomization. Patients’ clinical, surgical and oncological characteristics, such as BMI and method of reconstruction (DIEP or MS-TRAM) will be collected and their association with the primary and secondary outcomes will be tested in univariate analysis models to detect possible confounding factors. The effect of treatment groups will be studied next using an appropriate multivariate analysis model for each outcome while controlling for the possible confounding factors, if indicated, such as BMI and method of reconstruction. All statistical analyses will be performed using SAS software version 9.2 (SAS Inc, Cary, NC, USA). All tests will be two-sided, and significance will be set at *P* < 0.05.

The primary analysis will comprise mean postoperative opioid consumption during the first 48 hours postoperatively, which will be reported for the two groups as means (±SD). The two groups will be compared using the two-sample Student’s *t*-test or the Wilcoxon rank-sum test, depending on the results of the normality test. The secondary analysis will assess continuous outcomes, which will be reported for the two groups as means (±SD). The median and range will also be reported. The two groups will be compared using the two-sample Student’s *t*-test or the Wilcoxon rank-sum test, depending on the normality test.

Repeated-measures outcomes will be analysed to assess the effect of treatment (between-subject variable), time (within-subject variable) and a possible treatment × time interaction on outcomes measured at various time points. Time-to-event outcomes will be measured from the completion of surgery until the time of the event. Patients who do not experience the event at the time of the last follow-up will be censored. The number of censored events is expected to be low because both events (ambulation and bowel movement) are required to occur prior to the patient’s discharge from the hospital. Time-to-event outcomes will be analysed using the Kaplan-Meier method, and survival curves will be compared between the two groups using the logrank test.

### Sample size calculations

A retrospective review of 45 patients matched on key characteristics that underwent free MS-TRAM or DIEP breast reconstruction between January 2009 and March 2010 prior to the use of TAP block revealed a mean total opioid consumption of 9.2 mg ± 5.8 mg of intravenous morphine equivalent per patient. Using these preliminary data, we will require 29 patients per group to achieve 80% power to detect a 50% reduction in mean total opioid consumption between the control and study groups (9.2 vs. 4.6 mg, respectively) with group standard deviations of 5.8 mg and a significance level (Cronbach’s α) of 0.05 using a two-sided Wilcoxon rank-sum test assuming that the actual distribution is normal. From January to December 2010, approximately 100 patients underwent microsurgical breast reconstruction using abdominal tissue performed by the two participating surgeons (TZ and SOPH). It is anticipated that approximately 80% of these patients will be eligible and willing to participate in the study (approximately six or seven patients per month). A total of 58 patients will be enrolled into this proposed study, thus the estimated accrual time is approximately 9 to 12 months.

## Discussion

The use of peripheral nerve blocks has become increasingly popular over the past two decades. With the use of anatomical landmark-based techniques, novel types of nerve blocks have been designed to deliver effective and directed analgesia. Because a substantial component of the pain experienced by patients after abdominally based autologous tissue breast reconstruction is derived from the abdominal wall incision [3], our study is designed to evaluate the analgesic usefulness of TAP blockade inserted under direct vision in the abdominal donor sites following free MS-TRAM or DIEP flap harvest. In contrast to other studies that have investigated methods of improving postoperative analgesia with the use of a local anesthetic infusion catheter in the donor site following TRAM or DIEP flap reconstruction of the breast [11-15], our study is the first to apply a true peripheral nerve block under direct vision. Furthermore, in our previously published prospective cohort study, we found the use of the TAP block to be both safe and efficacious in postoperative pain control of the abdominal donor site [25]. There were no surgical complications associated with the placement of the catheter, and no incidence of local anesthetic toxicity was observed. The TAP catheters were well-tolerated by patients, and there was only one patient request for early TAP catheter removal. The intervention was cost-effective at approximately $100 per patient. However, this earlier study was limited by several shortcomings. First, there was an inherent bias in the use of historic controls as the comparison group. Second, the loss of patients to analysis due to major postoperative complications was relatively high for a small series (11.1% in the TAP block group and 17.5% in the control group). Third, the large range of opioids consumed in both groups indicates that responses to pain, as well as the need for analgesics, are highly variable and difficult to compare between individual patients.

The strength of our current double-blind, placebo-controlled RCT design lies in its innovative approach by applying a well-studied, anatomically based peripheral nerve block to the novel setting of autologous breast reconstruction using the most scientifically rigorous methodology [7,10,23]. By potentially decreasing the need for systemic opioids and their associated side effects, this trial will have the potential to improve both clinical care and health outcomes in patients undergoing abdominally based breast reconstruction in North America. Furthermore, this proposal represents innovative collaborative work between the reconstructive surgeons and anesthetists on this project. The anesthetists introduced the novel technique of TAP block as a form of postoperative analgesic method, but it is the surgeons who are most familiar with the anatomical planes of the anterior abdominal wall. Another advantage of using the TAP block in the donor site following a free TRAM or DIEP harvest is that the TAP catheters can be inserted into the triangle of Petit under direct vision. In addition, Clinical equipoise exists in this trial because no prior RCTs have evaluated the efficacy of the TAP block in improving pain symptomatology following abdominally based, autologous tissue breast reconstruction.

### Risks and benefits

There are potential surgical risks associated with the insertion of a TAP catheter into the transversus abdominus plane. The minimal access incision over the triangle of Petit will be repaired using the same technique as that used for the rectus sheath repair, which is a part of the autologous tissue reconstruction procedure. Furthermore, the insertion of the TAP catheters under direct vision should theoretically be safer than the blinded technique or ultrasound-guided technique that is currently practiced by some anesthetists. To safeguard against possible bupivacaine toxicity, all patients with cardiac or liver disease will be excluded from the study. In addition, a member of the pain service as well as the study coordinator will check for early signs of central nervous system toxicity, such as perioral numbness and tingling, restlessness, anxiety, lightheadedness, metallic taste, tinnitus, dizziness, blurred vision, tremors, drowsiness and incoherent speech at 15 minutes, 30 minutes and 1 hour following each TAP bolus dose delivery. It has been found in previous studies that peak serum concentrations of LA in TAP blocks in most patients occurred at 30 minutes after injection [26].

### Limitations and strengths

To avoid having a group of patients who will receive only the placebo fluid through the TAP catheter, we contemplated the use of a cross-over study design. Unfortunately, this design is not feasible in our study, because our local anesthetic, bupivacaine, is long-acting and requires at least a 12-hour washout period. Because the primary objective of our trial is to measure opioid consumption in the first 48 hours postoperatively, it is not possible to build into our study design a 12-hour washout period between administration of the study and placebo agents.

The proposed TAP block, double-blind, placebo-controlled RCT represents the first RCT to assess the efficacy of the TAP block in improving pain symptomatology following abdominally based, autologous tissue breast reconstruction. The results of this trial have the potential to improve postoperative analgesic control for breast cancer patients undergoing this common type of major reconstructive surgery.

### Trial status

The TAP Block RCT study began enrolment in September 2011 at Toronto General Hospital. At the date of manuscript acceptance, the trial has been enrolling participants for 24 months and close to accrual of the required sample. A total of 129 patients were eligible for participation, and 86 patients have been enrolled into the trial. Eligible patients who declined to participate were similar in all respects to the patients who agreed to participate. About 50% of the patients who declined to participate in the study did not provide a reason, 30% were ambivalent about being randomized and unsure about participation in the study and 10% to 20% indicated that they were too anxious and upset to consider entering a trial in addition to undergoing major reconstructive surgery. To date, there have been no major TAP catheter-related surgical complications and no instances of local anesthetic toxicity However, three patients experienced blocked TAP catheters that could not be used to deliver bolus injections, and two study patients received bupivacaine instead of the study medication in error.

## Abbreviations

DIEP: Deep inferior epigastric perforator flap reconstruction; LA: Local anaesthetic; MS-TRAM: Muscle-sparing transversus abdominus myocutaneous flap reconstruction; OR: Operating room; PCA: Patient-controlled anaesthesia; QoR: Quality of recovery questionnaire; RCT: Randomized controlled trial; TAP: Transversus abdominis plane.

## Competing interests

The authors do not have any competing interests, financial or otherwise, to report.

## Authors’ contributions

TZ conceived of and will co-lead the proposed trial, screen eligible patients, insert TAP catheters, participate in the analysis and interpretation of data and draft the manuscripts. CS will co-lead the proposed trial, supervise study medication schedules, participate in the analysis and interpretation of data and draft the manuscripts. SOPH will contribute to enrolment of patients, insert TAP catheters, participate in the analysis and interpretation of data and draft the manuscripts. AO will contribute to enrolment of patients, insert TAP catheters and participate in the analysis and interpretation of data. HC will contribute expertise in conducting the trial, monitor study medications and participate in manuscript drafts. SAM will contribute expertise in conducting the trial, monitor study medications and participate in the analysis and interpretation of data as well as in manuscript drafts. MO will screen and enrol eligible study participants, monitor study medication delivery and collect and summarize inpatient and hospital discharge data and participate in the analysis and interpretation of data. KB will screen and enrol eligible study participants, collect baseline data, fax study medication sheets to the pharmacy for randomization and participate in the analysis and interpretation of data. SB will be responsible for data entry and cleaning and participate in the analysis and interpretation of data and in manuscript drafts. All authors have read and approved the final manuscript.

## Authors’ information

TZ holds a Career Development Award from the American Society of Clinical Oncology. SOPH holds the Wharton Chair in Reconstructive Plastic Surgery and is chief of the Division of Plastic Surgery, Department of Surgery, University Health Network. SOPH and TZ are supported by grant funding from the Canadian Breast Cancer Foundation and the Canadian Institutes of Health Research. SAM is medical director of the Perioperative Blood Conservation Program at the University Health Network. HC is director of the Pain Research Unit at Toronto General Hospital. The other coauthors do not have any information to report.
